# Authentic collaboration in research: partnering with people with lived experience

**DOI:** 10.1038/s41390-025-03824-5

**Published:** 2025-02-06

**Authors:** Natasha Garrity, Kylie Facer, Rod Hunt, Shannon Olivey, Sophie Marmont, Nadia Badawi, Melinda Cruz, Sarah McIntyre

**Affiliations:** 1 Cerebral Palsy Alliance Research Institute, Sydney Medical School, University of Sydney, Sydney, NSW Australia; 2 CP Quest, Cerebral Palsy Alliance, Sydney, NSW Australia; 3 Paediatrics Education & Research, Monash University, Clayton, VIC Australia; 4 NICU Lived Network, Sydney, Australia

Historically research has been initiated, designed, and conducted by researchers and clinicians based on their interests, expertise, literature informed “next steps” and pragmatically—funding opportunities. The world of developmental disability has been no different. People with the condition being researched were traditionally limited to being participants of the clinical trials that were decided on by researchers and clinicians, participating with hopes of it benefitting them. It is only in the last few decades that people with lived experience were “permitted” to attend conferences, helped prioritise research and have been involved as partners in research on the conditions they had a genuine understanding of.

While there were many reasons and justifications for this,^1^ in recent years we now actively recognise the importance of the ‘consumer/lived experience’ voice in ensuring we are answering questions that are important to people who experience the condition, in a way that is ethical and acceptable to both families and the individuals with the condition. Since the late 2000’s researchers’ awareness of the value of lived experience has been shifting, moving toward a complimentary partnership. People with lived experience (commonly being referred to as consumers) are now demanding or being invited to have a seat at the table and a role in decision making around what gets researched, how it gets researched and how the results of the research get implemented and shared. Overall, this has had a major positive impact on both the scientific community and the communities that ultimately benefit from the work.^2^

However, in our enthusiasm we have adopted language that might not be fit for purpose. ‘Consumer involvement’ is the term currently being used by the National Health and Medical Research Council (NHMRC). The term ‘consumer’ came from a movement away from the term ‘patients’. This was believed to be more reflective of their ‘new’ role as partners in their own health care instead of being passive recipients. Furthermore, the term “consumer” in health has been labelled as an imperfect fit in the past^1^ and there are groups of people who have strong thoughts about the word “consumer” both in research and a therapy/services context.^3^

Many people with lived experience find the term ‘consumer’ in the context of health and disability uncomfortable. While it does make some logical sense as they are the ones that are or will be ‘consuming’ the therapy/service/treatment, the word ‘consumer” in its traditional business use also implies choice. For many people with lived experience of a complicated condition, choice is not something they feel they have the luxury of. For many parents in particular, choice is exactly what they felt they lacked. Often in the early life of a child with a condition like cerebral palsy, it is at best emotional and at worst traumatic. Parents are faced with an uncertain future, and often told by doctors or other health professionals, words to the effect of ‘your child has XYZ, in order for your child to have the best quality of life/survive, ABC should be done as soon as possible.’ In fact the word “quality” is very subjective and imparts a value judgement that may or may not be shared by people with lived experience.

This theme continues for the people with cerebral palsy (CP), where therapy is not an ‘option’ but a necessary component of life to achieve the most independence and self-determination possible. There are many anecdotes within the disability community that equate to the following: You can choose not to receive therapy/treatments or try different kinds of interventions, but the lack of therapy, or trying ineffective therapy can lead to health complications, and a loss of mobility and independence over time. This is particularly the case for children with ambulant CP.^4,5^ In this context, the choice implied by the word consumer seems redundant.^6,7^ In addition, while “consumer” can be seen as an alternative to ‘patient’, there is still a certain lack of power. We can only consume from what is available to us, which varies greatly depending on where we live.

From the first opportunity, people with lived experience are often keen to help researchers understand more about their condition and improve the experience of services and other treatments for future parents and people with CP. Collaborating and partnering on research projects is one way to achieve this. First-time collaborators are often taken aback by academic jargon, and care should be taken with language choices to make people with lived experience feel as comfortable as possible. The phrase ‘consumer involvement’ has become commonplace to represent this collaboration and partnership and to easily distinguish between those on the research team with lived experience and those participating in the study.^8^ However, just as ‘consumer’ is a term that many people with lived experience do not agree with regarding services, for the same reasons, so is the term ‘consumer’ or ‘consumer involvement’ in research. The founding members of CP Quest (Researchers and Community Together) were strongly against the use of this term. The alternative term of a ‘Research Partner’ was agreed upon by our community. It is often preferred by people with lived experience as it invokes images of an active partnership, working together, with complimentary roles and skills sets to improve the quality of research. Many find it an empowering point of difference in the medical world where the terms “patient”, “carer” and now “consumer” are the norm.^9^

In most chronic conditions, the language surrounding the condition and treatments for an individual are often a sensitive subject. For the best results in winning the trust of the community you are working with, it’s best to take a moment to ‘read the room’. Use the language that is the most acceptable for the community you are working with, that is—give them options and/or ask them what term they prefer. Often once a term has been agreed upon, communication is easier and more open, with trust more easily built. For this reason, it is critical that researchers and clinicians continue to be aware of the impact their language has on those they are striving to help. Being open to change as language evolves ultimately will benefit all.
